# Paternal sleep deprivation and cardiac dysfunction in offspring: A perspective of myocardial hypertrophy

**DOI:** 10.1113/EP093212

**Published:** 2026-04-09

**Authors:** Yingying Zhang, Leyao Wang, Jie Wang, Yun Pan, Wen Sun, Zhiwei Zou, Wenlin Bai, Ruiling Fang, Yue Zhang, Ping Zhang, Yuanbao Zhang, Lijian Lei, Wenping Zhang

**Affiliations:** ^1^ Shanxi Key Laboratory of Environmental Health Impairment and Prevention, NHC Key Laboratory of Pneumoconiosis, MOE Key Laboratory of Coal Environmental Pathogenicity and Prevention Shanxi Medical University Taiyuan Shanxi China; ^2^ Beijing Key Laboratory of Occupational Safety and Health, Institute of Urban Safety and Environmental Science Beijing Academy of Science and Technology Beijing China

**Keywords:** acute sleep deprivation, cardiac dysfunction, offspring, paternal

## Abstract

The objective of this work is to assess the effects and potential process of paternal acute sleep deprivation on cardiac function in their offspring. Male and female C57BL/6N mice aged 8–9 weeks (*n* = 4–5 mice/group) were used. Male mice were selected to construct an acute sleep deprivation model using the modified multiple platform method (MMPM) and mated with normal female mice. After gestation, the weight change of offspring mice was observed and recorded. At the time of 10 weeks, the changes of cardiac function were evaluated by blood pressure, heart rate and echocardiography; cardiac histology and cytopathological changes were observed by haematoxylin–eosin staining; and the molecular mechanism induced by acute paternal sleep deprivation were further elucidated by molecular biological techniques. In the acute sleep deprivation group, male offspring showed significant cardiac hypertrophy, whereas female offspring did not. mRNA levels of GATA binding protein 4 (GATA4) (*P* < 0.01) and mRNA levels of classic hypertrophic genes, such as atrial natriuretic peptide (ANP) (*P* < 0.05) and brain natriuretic peptide (BNP) (*P* < 0.05), increased significantly in the hearts of the acute sleep deprivation group. Paternal acute sleep deprivation may lead to cardiac dysfunction in offspring through cardiac hypertrophy.

## Abstract (highlights)

- **What is the central question of this study?**
  What are the effects and potential mechanism of paternal acute sleep deprivation on cardiac function in their offspring.
- **What is the main finding and its importance?**
  Paternal sleep deprivation can cause sex‐specific cardiac dysfunction in offspring, with males exhibiting more pronounced effects manifested as myocardial hypertrophy.

## 1 INTRODUCTION

About one‐third of a person's life is spent sleeping, a physiological process that is vital to maintaining proper body function and promoting health (Chaput et al., 2023). With the development of society, sleep deprivation is gradually becoming a global public health problem. According to the Centers for Disease Control and Prevention, 35% of Americans sleep every night well below the 7–9 h recommended by most experts based on existing findings, and this is classified as a public epidemic (Gonzalez & Tyminski, 2020). Sleep deprivation is considered an increasingly common condition inherent in modern society (Tobaldini et al., 2014) and is one of the causes of many significant health risks (Liew & Aung, 2021). Long‐term sleep deprivation not only has a profound impact on one's health, leading to a decline in the body's immunity, increasing the risk of metabolic diseases, cancer, mental illness, etc. (Borbély et al., 2018; Kim et al., 2015), but also has an impact on the health of their offspring (Harskamp‐van Ginkel et al., 2020; Yao et al., 2022).

Numerous studies have shown that maternal sleep deprivation during pregnancy increases the risk of obesity (Harskamp‐van Ginkel et al., 2020) and reduces the reproductive capacity of their offspring (Alvarenga et al., 2013); there are also effects on cardiac function in their offspring and differences between males and females (Lima et al., 2014). In addition, sleep deprivation during pregnancy in mothers also impairs learning and memory in their offspring, leading to cognitive dysfunction. These findings suggest that the effects of maternal sleep deprivation on offspring are relatively mature, but little attention has been paid to whether there is an important paternal role in the genetic process.

Currently, sleep deprivation has emerged as a serious public health concern associated with cardiovascular disease, and sleep duration has emerged as an indicator to predict cardiovascular disease outcomes (Pasetes et al., 2023). The heart is the central organ of the circulatory system, and its normal development is a complex process that is essential for maintaining human life (Cui et al., 2019). Mammalian cardiomyocytes have a certain ability to divide at their embryonic and primary stages, but when they reach adulthood, most cardiomyocytes lose their ability to proliferate (Chen et al., 2021). Thereafter, in order to meet increased workload demands, the heart undergoes changes under various stress conditions (Samak et al., 2016), with increased cardiomyocyte size and cardiac hypertrophy. It has been shown that maternal high salt intake during pregnancy causes cardiac hypertrophy in adult male offspring (Alves‐Rodrigues et al., 2013), and maternal high fat diet is also associated with increased cardiovascular risk in offspring (De Jong et al., 2018). There is a paucity of scientific research on how paternal exposure affects cardiac function and cardiac hypertrophy in offspring, and an even greater paucity of research addressing the underlying molecular mechanisms behind this effect.

In this study, male C57BL/6N mice were used to establish an acute sleep deprivation model, then mated with normal females to evaluate cardiac function changes in offspring, probing paternal impact mechanisms in order to address the following questions: Does sleep deprivation in parents leads to changes in cardiac function in adult offspring? If so, what are the associated biological alterations and molecular mechanisms?

## 2 METHODS

### 2.1 Ethical approval

All animal experiments were conducted in strict accordance with the guidelines and regulations of the Shanxi Medical University Animal Care and Use Committee (Ethical Approval No. 2020GLL042) and complied with the ARRIVE (Animals in Research: Reporting In Vivo Experiments) guidelines. Procedures were designed to minimize suffering and humane endpoints as defined by ethical guidelines. All efforts were made to reduce the number of animals used, aligning with the 3Rs principle (Replacement, Reduction, Refinement).

Male and female C57BL/6N mice aged 8–9 weeks were purchased from Beijing Vital River Laboratory Animal Technology Co., Ltd (Beijing China; Permit Number: SCXK (Jing) 2021‐0006) and maintained under a 12h‐light–dark cycle at room temperature (24 ± 2°C) and humidity (50 ± 5%) with free access to water and food; the experiment was started after 1 week of adaptation. After the experiment, the mice were sacrificed via cervical dislocation. The heart samples were excised, weighed, quickly frozen in liquid nitrogen and stored at −80°C for further analyses.

### 2.2 Animal grouping and treatment

After mice were acclimatized to feeding for 1 week, female mice continued normal feeding. Male mice were randomly divided into a control group (CON group) and an acute sleep deprivation group (SD group) by a random number table according to body weight. The SD group was treated with the modified multi‐platform method (MMPM) of sleep deprivation by small platform acute sleep deprivation for 72 h. The CON group was placed on a large platform. After the experiment, sperm was collected from the epididymis of some of the male mice, while others were mated with female mice at a 2:1 ratio; the following morning, vaginal plugs were examined to confirm pregnancy. Offspring mice were weighed on postnatal days 1, 7, 14 and 21 and weeks 4, 6, 8 and 10; at the end of week 10, some underwent heart rate and blood pressure measurement and echocardiography, and from some heart tissues were collected for histopathological observation and RT‐qPCR.

### 2.3 Construction of mouse SD model by MMPM

In this study, we established the SD mouse model using the MMPM, which deprives mice of rapid eye movement sleep. The MMPM used a box of 40 cm × 30 cm × 30 cm, and 12 cylindrical small platforms with a diameter of 3 cm and a height of 20 cm were placed internally (Liu et al., 2022). The mice could move freely between platforms. Water was added to the box to 1 cm below the small platform, and the water temperature was controlled at about 24°C. The water was changed once a day to keep it clean, and the water tank was placed with a mesh cap above, food and drinking bottles were placed above the mesh cap, and the mice had free access to water and food during the experiment. The principle of the MMPM model is that mice fall easily into the water as they enter sleep due to muscle hypotonia, forcing them to climb back to the platform to stay awake. The CON group used a large platform tank, and the difference between these two tanks was that the platform in the control group was large enough that mice could rest and sleep without falling into the water. Two days before the start of the experiment, mice were placed under the same conditions as the formal experiment daily for 2 h environmental adaptation training to avoid stress affecting the accuracy of the experimental results.

### 2.4 Sperm viability assay

To detect the viability of spermatozoa in the epididymal tails of paternal mice, 2 mL of medium was added to a 5 mL EP tube and placed in a 37°C water bath for preheating. The removed epididymal tails were quickly placed in the EP tube and clipped to free the spermatozoa and make a sperm suspension. The large uncut tissue was filtered by a filter membrane.

A drop of sperm suspension was taken on the sperm counting plate, covered with a coverslip, and the number of sperms inside every 20 compartments was recorded under the microscope, videotaped for 10 s. Sperm motility (%) = Total number of live sperms/(number of live sperms + number of dead sperms) × 100%.

### 2.5 Sperm morphology observation

A small drop of the above sperm suspension was taken for a smear, dried, fixed in methanol for 5 min, then stained with 1–2% eosin for 1 h, rinsed, dried and examined microscopically. The number of malformed spermatozoa was counted in at least 1000 intact spermatozoa, and the sperm malformation rate (%) = number of malformed spermatozoa/total number of spermatozoa × 100%.

### 2.6 Enzyme‐linked immunosorbent assay

Serum samples were collected from parental mice, and serum testosterone (Wuhan Fine Biotech Co., Ltd, Wuhan, China) and corticosterone (Jiangsu Meimian Industrial Co., Ltd, Yancheng, China) levels were assayed using an enzyme‐linked immunosorbent assay kit according to the manufacturer's instructions. The absorbance (OD value) was measured at 450 nm using an enzyme labelling instrument (TECAN, Männedorf, Switzerland), and a standard curve was plotted according to the OD value and concentration of the standards, and the testosterone and corticosterone concentration in the samples was calculated.

### 2.7 Echocardiography testing

Cardiac function was assessed using a Vevo 2100 imaging system (Visualsonics, Toronto, Canada) equipped with a high‐frequency (30 MHz) linear array transducer. First, the left anterior chest of the mice was depilated, and then the mice were anaesthetized with an anaesthesia machine; they were placed supine on an ultrasound operating table at a constant temperature of 37°C, and two‐dimensional and M‐mode ultrasound images were collected under continuous low‐concentration anaesthesia. Cardiac function parameters, including isovolumic contraction time (IVCT, ms), isovolumic relaxation time (IVRT, ms), left ventricular peak early diastolic velocity/left ventricular peak late diastolic velocity (*E*/*A*), left ventricular anterior end‐diastolic wall thickness (LVAW;d, mm), left ventricular anterior end‐systolic wall thickness (LVAW;s, mm), left ventricular end diastolic diameter (LVID;d, mm), left ventricular end systolic diameter (LVID;s, mm), left ventricular posterior end‐diastolic wall thickness (LVPW;d, mm), left ventricular posterior end‐systolic wall thickness (LVPW;s, mm), left ventricular mass (LV mass AW, mg), corrected left ventricular mass (LV AW (corrected), mg), left ventricular diastolic volume (LV Vol;d, µL), and left ventricular systolic volume (LV Vol;s, µL), were measured to assess cardiac function in mice. In addition, ejection fraction (EF), shortening fraction (FS) and Tei index were calculated.

### 2.8 Haematoxylin and eosin staining

After decapitation, the heart tissues of the offspring mice were collected, and the blood in the cavity was washed with 0.9% saline and soaked in 10% formalin overnight at room temperature. The following day, the tissues were removed and embedded in paraffin, and then the tissues were cut into sections with a thickness of 5–6 µm for haematoxylin and eosin (HE) staining. Histopathological changes such as myocardial thickness and cardiomyocyte size were observed by light microscopy.

### 2.9 Reverse transcription real‐time quantitative polymerase chain reaction (RT‐qPCR)

Total RNA (TransGen Biotech, Beijing, China) was extracted from heart tissue of 10‐week‐old offspring mice using Trizol reagent. Thirty milligrams of heart tissue was weighed, homogenized with 300 µL Trizol lysate, and centrifuged with chloroform and isopropanol. The pellet was washed with 75% alcohol, then dissolved with an appropriate amount of nuclease‐free water, and RNA concentration was determined using an ultramicrospectrophotometer (Thermo Fisher Scientific, Waltham, MA, USA). RNA was reverse transcribed into cDNA using a reverse transcription kit (TransGen Biotech). Finally, β‐actin was used as an internal reference, and the expression of related genes was relatively quantitatively detected using a fluorescence quantitative PCR instrument (Bio‐Rad Laboratories, Hercules, CA, USA), and relative gene levels were calculated using the $2^{-\Delta \Delta C_{t}}$ method.

### 2.10 Statistical analysis

Data entry and statistical analysis were performed by SPSS 26.0 software (IBM Corp., Armonk, NY, USA) and plots were performed using GraphPad Prism 8.0 (GraphPad Software, San Diego, CA, USA). The obtained experimental results were expressed as means ± standard deviation (SD). Data were compared between control and acute sleep deprivation groups using Student's two independent samples *t*‐test. *P* < 0.05 was considered statistically significant.

## 3 RESULTS

### 3.1 Effects of paternal sleep deprivation on reproductive health in mice

**FIGURE 1:**
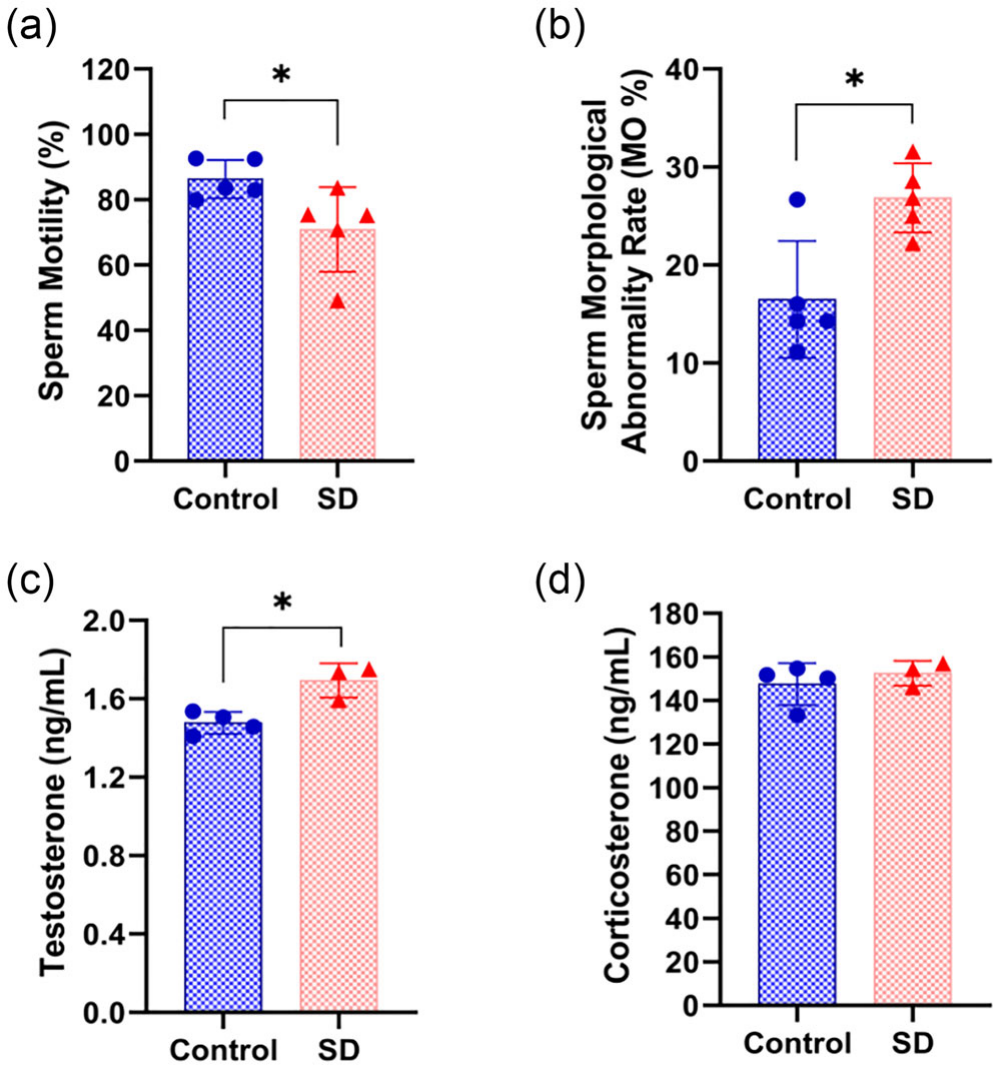
Evaluation of reproductive health in mice after sleep deprivation. (a) Microscopic observation of sperm motility in the epididymal tail of mice in the control group and SD group (*n* = 5 mice/group). (b) Observed sperm morphology in both groups using Haematoxylin and eosin staining (*n *= 5 mice/group). (c) Measured serum testosterone concentrations in control mice and SD mice via ELISA (*n* = 3–4 mice/group). (d) Measured serum corticosterone concentrations in control mice and SD mice via ELISA. *n* = 3–4 mice/group. Data are expressed as means ± SD and were analysed using an independent samples *t*‐test. *P < 0.05 compared with control.

To assess the reproductive toxicity of sleep deprivation in male mice, we examined sperm quality and serum testosterone levels in paternal mice. The study revealed that compared to the control group, sperm motility levels were significantly reduced in male mice subjected to sleep deprivation (*P *< 0.05, Figure 1a); simultaneously, the sperm abnormality rate in the sleep deprivation group was significantly higher than that in the normal control group (*P* < 0.05, Figure 1b). Serum testosterone levels in the sleep‐deprived group were significantly elevated (*P *< 0.05, Figure 1c), suggesting impaired male reproductive hormone function. These findings indicate that sleep deprivation may adversely affect male reproductive health. To rule out the influence of stress as a potential confounding factor, we measured serum corticosterone levels. Compared to the normal sleep control group, corticosterone levels in sleep‐deprived mice showed no significant change, indicating that our sleep deprivation method did not induce substantial alterations in stress status (*P* > 0.05, Figure 1d).

### 3.2 Effect of paternal sleep deprivation on basic developmental indicators in offspring

**FIGURE 2:**
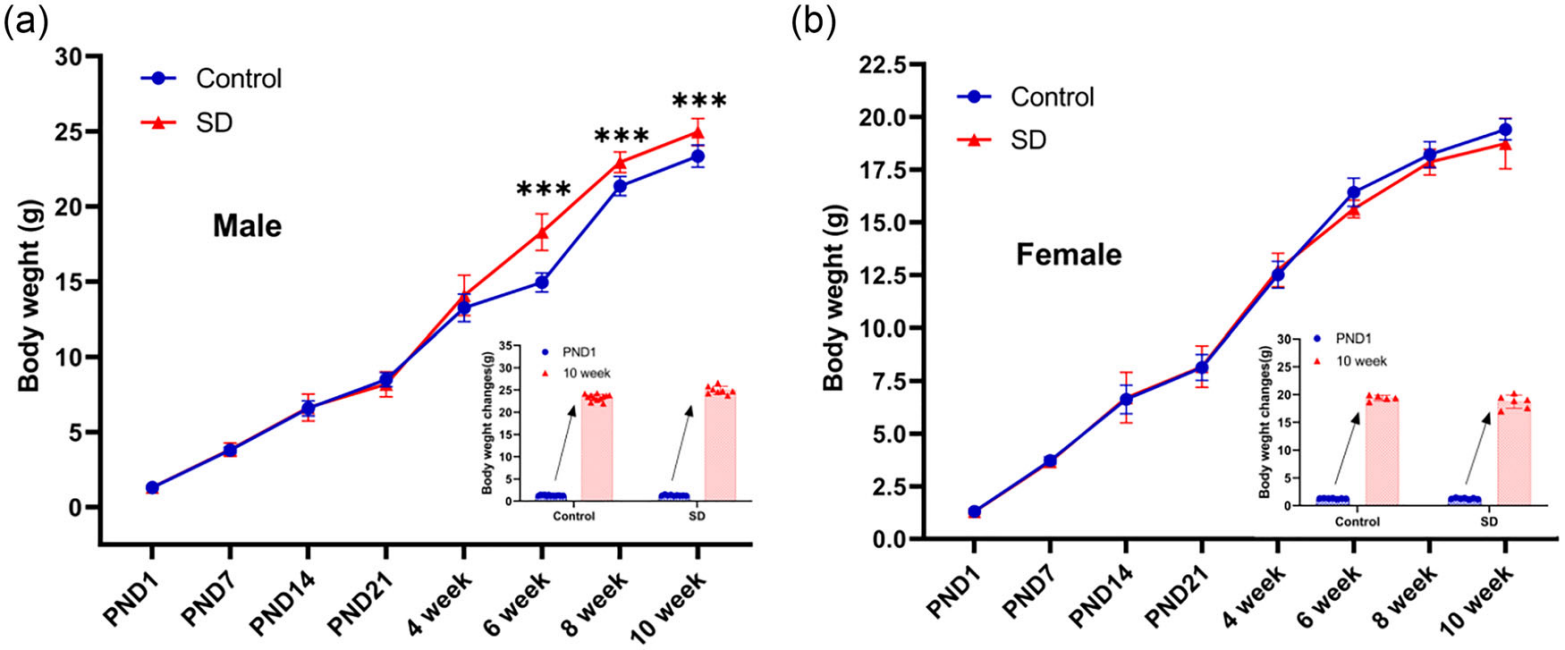
Body weight in offspring mice before 10 weeks. (a) Body weight changes in male offspring mice. *n* = 8–13 mice/group. (b) Body weight changes in female offspring mice. *n* = 5–7 mice/group. Data are expressed as means ± SD and were analysed using an independent samples *t*‐test. ***P < 0.001 compared with control.

Following the end of parental sleep deprivation, we mated each group of mice with normal females. Birth weight during postnatal growth and development of offspring mice was first followed up and monitored, and the results are shown in Figure 2. Compared with the control group, offspring male mice had significantly higher body weights at 6, 8 and 10 weeks after birth (*P* < 0.001), and there was no difference in female offspring mice (*P* > 0.05). In addition, there were no significant changes in coat colour, ear margin and limbs, and no significant abnormalities in drinking water, eating and mental status during the growth of mice in both groups.

### 3.3 Effect of paternal sleep deprivation on cardiac function in offspring

**FIGURE 3:**
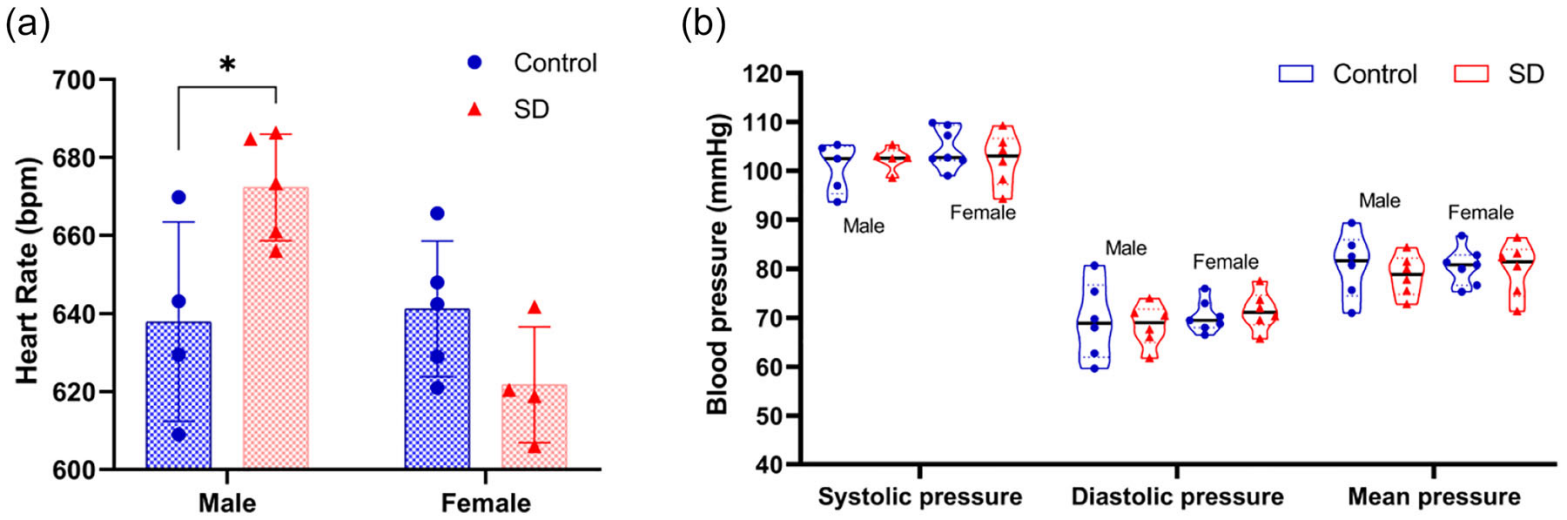
Effects of paternal sleep deprivation on heart rate and blood pressure in adult mice before pregnancy. Heart rate (HR, bpm) (a) and blood pressure (BP, mmHg) (b) in male and female offspring mice from the control group and sleep‐deprived group. *n* = 4–7 mice/group; data are expressed as means ± SD and were analysed using an independent samples *t*‐test. *P < 0.05 compared with control.

**FIGURE 4:**
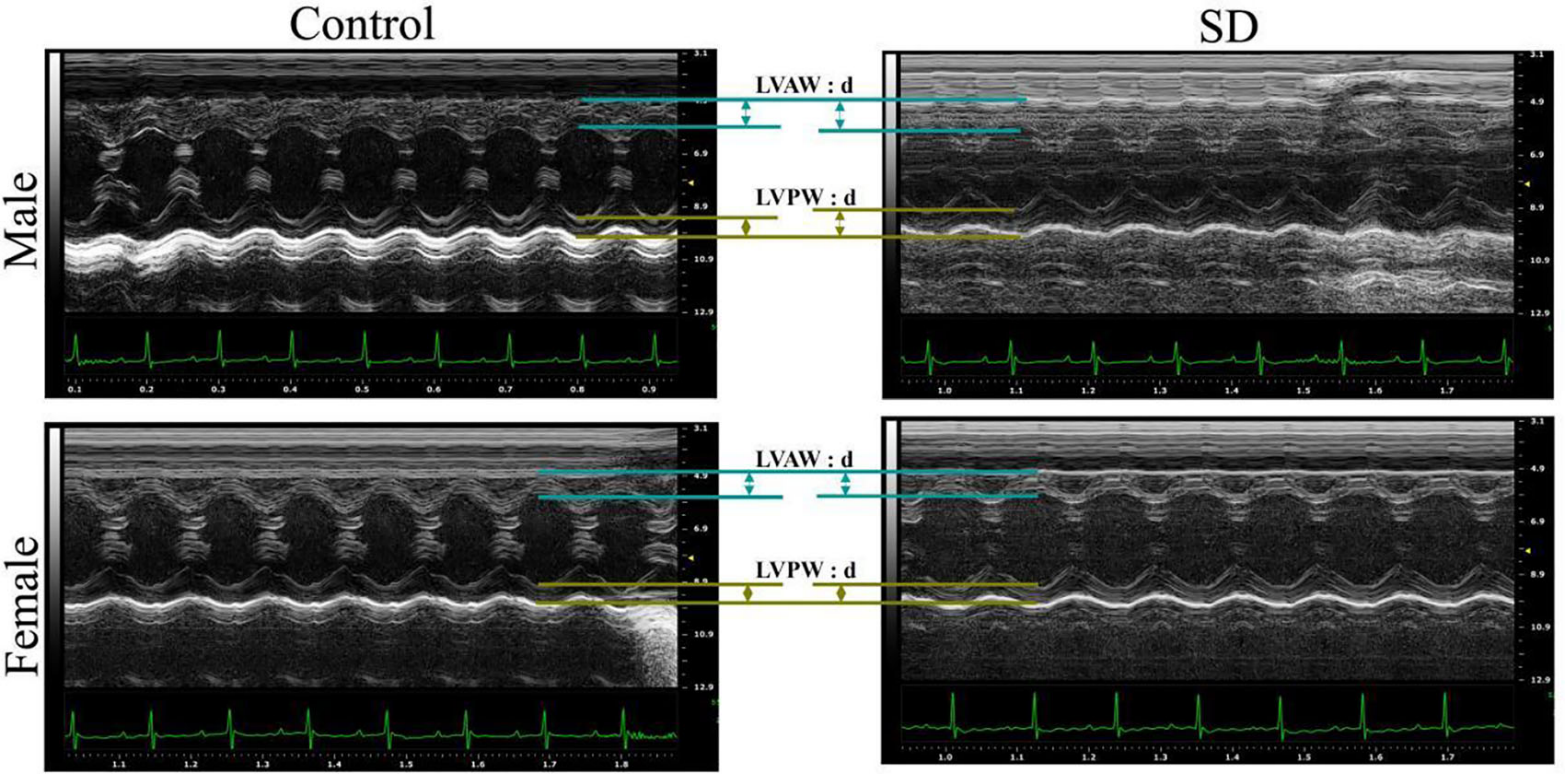
Representative echocardiograms of adult mice of the offspring. End‐systolic posterior wall thickness shown by arrows in the figure.

In order to investigate the effect of paternal sleep deprivation on cardiac function in offspring adult mice, we selected 10‐week‐old offspring mice for heart rate and blood pressure measurement and echocardiography. The results of heart rate and blood pressure tests showed that sleep deprivation of fathers before pregnancy was able to cause an increase in heart rate in offspring male mice (Figure 3a), and there was no significant difference in blood pressure in offspring mice (Figure 3b). Echocardiography‐related parameters of 10‐week‐old offspring mice showed that sleep deprivation of the father before pregnancy caused significant changes in systolic and diastolic function in offspring mice, and some indicators showed sex differences. Compared with the control group, EF and FS were significantly decreased in offspring male and female mice (*P *< 0.05), suggesting systolic dysfunction; IVRT time was prolonged in offspring male mice (*P *< 0.05), suggesting diastolic dysfunction, LVAW;d and LVPW;d were significantly increased in offspring male mice (*P *< 0.05), suggesting ventricular wall hypertrophy and decreased cardiac function in mice, and Tei, an indicator of the overall state of cardiac function, was also significantly increased in offspring male mice (*P *< 0.01) (Table 1). Decreased systolic function and thickening of the ventricular wall were also seen in the echocardiograms of the 10‐week‐old offspring mice (Figure 4), which, as seen in the male offspring, was significantly increased.

**TABLE 1:** Effect of paternal sleep deprivation on cardiac function in offspring mice.

Sex/parameter	Male		Female	
	Control	SD	Control	SD
IVCT (ms)	10.15 ± 4.00	11.83 ± 1.84	10.04 ± 2.39	11.39 ± 1.24
IVRT (ms)	10.94 ± 2.86	15.19 ± 2.76^*^	14.05 ± 1.74	13.21 ± 2.50
Tei	0.43 ± 0.07	0.63 ± 0.10 ^*^	0.54 ± 0.07	0.59 ± 0.12
*E*/*A*	1.39 ± 0.15	1.32 ± 0.10	1.53 ± 0.32	1.44 ± 0.37
LVAW;d (mm)	0.58 ± 0.08	0.69 ± 0.06 ^*^	0.56 ± 0.05	0.60 ± 0.13
LVAW;s (mm)	1.04 ± 0.10	1.02 ± 0.12	1.08 ± 0.07	0.96 ± 0.16
LVID;d (mm)	3.62 ± 0.24	3.74 ± 0.34	3.67 ± 0.29	3.75 ± 0.26
LVID;s (mm)	2.31 ± 0.40	2.65 ± 0.23	2.19 ± 0.39	2.59 ± 0.12
LVPW;d (mm)	0.60 ± 0.06	0.76 ± 0.04 ^*^	0.61 ± 0.07	0.61 ± 0.05
LVPW;s (mm)	1.03 ± 0.10	1.13 ± 0.03	1.12 ± 0.18	1.04 ± 0.09
EF (%)	72.73 ± 5.45	60.44 ± 3.81 ^*^	71.71 ± 8.28	57.97 ± 4.51 ^*^
FS (%)	41.27 ± 4.52	31.69 ± 2.50^*^	40.58 ± 6.44	27.94 ± 4.02^*^
LV mass AW (mg)	75.74 ± 9.54	85.76 ± 14.46	67.01 ± 9.44	68.82 ± 7.02
LV mass AW (corrected) (mg)	58.54 ± 6.42	68.61 ± 11.57	54.75 ± 7.84	57.59 ± 7.47
LV Vol;d	63.22 ± 13.77	60.26 ± 12.95	57.51 ± 10.70	60.29 ± 10.62
LV Vol;s	19.04 ± 8.18	24.16 ± 7.00	18.36 ± 7.93	24.45 ± 2.68

* *P* < 0.05 compared with control. Abbreviations: *E*/*A*, left ventricular early diastolic blood flow peak velocity/left ventricular late diastolic blood flow peak velocity; EF, ejection fraction; FS, left ventricular fractional shortening; IVCT, isovolumic contraction time; IVRT, isovolumic relaxation time; LV mass AW, left ventricular mass; LV mass AW (corrected), corrected left ventricular mass; LV Vol;d, left ventricular diastolic volume; LV Vol;s, left ventricular systolic volume; LVAW;d, left ventricular end‐diastolic anterior wall thickness; LVAW;s, left ventricular end‐systolic anterior wall thickness; LVID;d, left ventricular end‐diastolic diameter; LVID;s, left ventricular end‐systolic diameter; LVPW;d, left ventricular end‐diastolic posterior wall thickness; LVPW;s, left ventricular end‐systolic posterior wall thickness; Tei, myocardial work index.

### 3.4 Effect of paternal sleep deprivation on cardiac morphology in offspring

**FIGURE 5:**
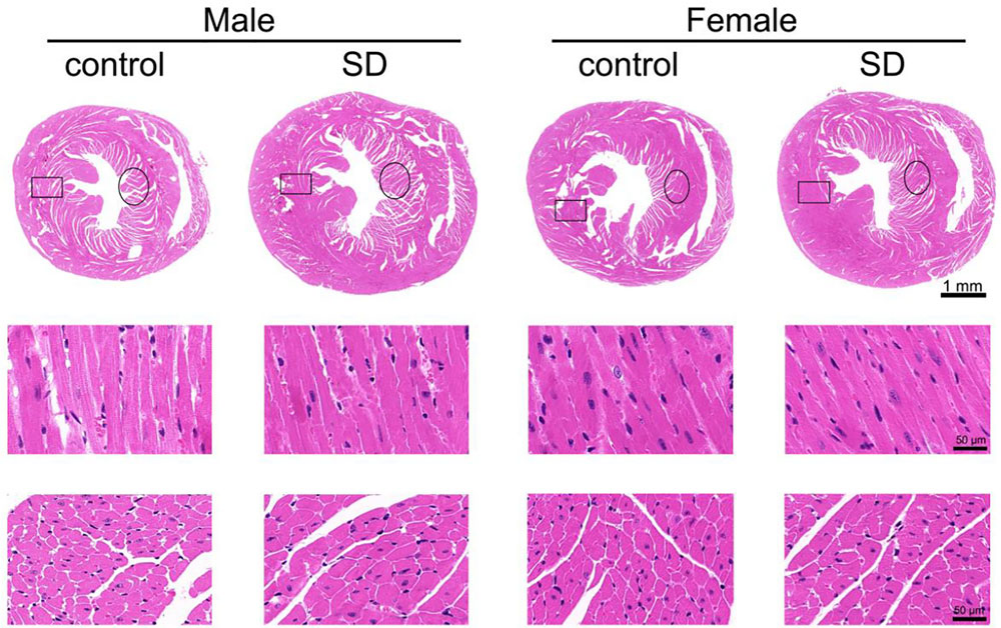
Representative histopathological images of offspring mouse hearts stained with HE. Longitudinal and transverse sections of hearts from male and female offspring mice. Scale bar = 1 mm or 50 µm.

We collected heart tissue samples from offspring mice, which were subsequently processed for HE staining to observe and analyse morphological changes in the heart. Paternal sleep deprivation induced significant cardiac hypertrophy in male offspring, but not in female offspring (Figure 5). We further amplified the myocardium and observed the morphological changes of cardiomyocytes. The results showed that sleep deprivation before pregnancy caused cardiomyocyte enlargement in offspring male mice, but there was no significant difference in female offspring.

### 3.5 Effects of paternal sleep deprivation on atrial natriuretic peptide and brain natriuretic peptide expression in the hearts of offspring

**FIGURE 6:**
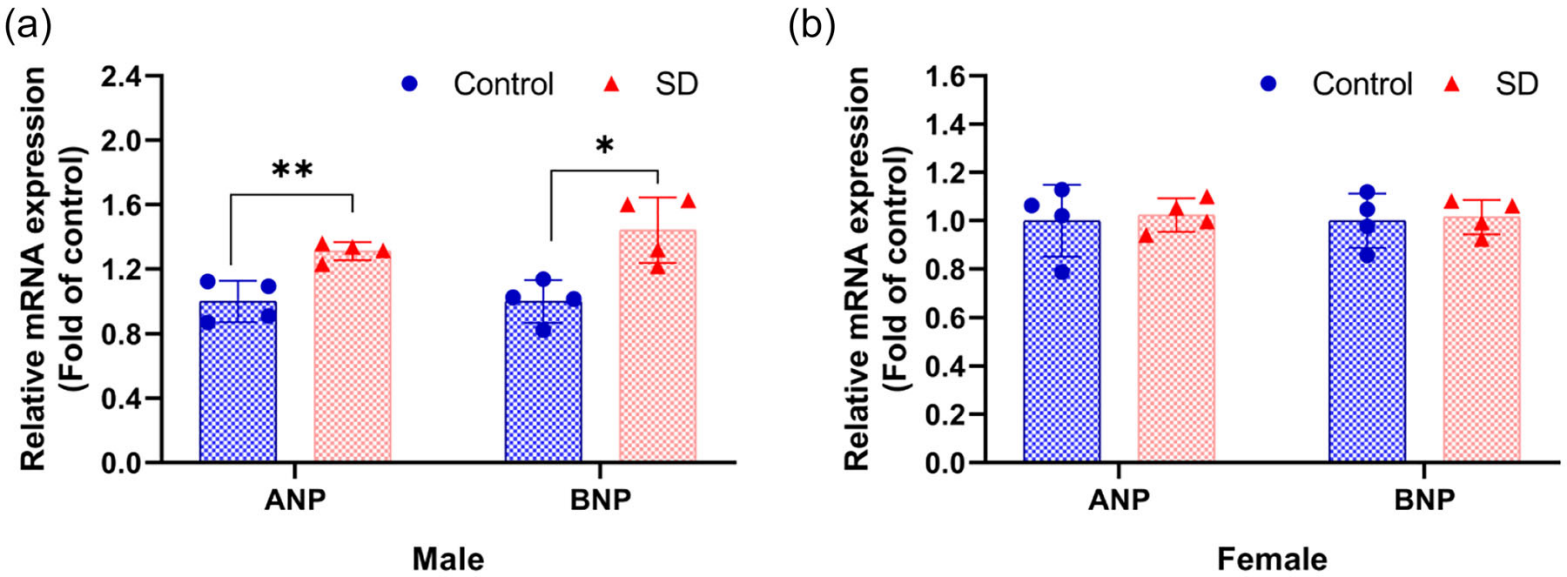
Effects of paternal sleep deprivation on mRNA expression of ANP and BNP in the heart of offspring. RT‐qPCR detection of ANP and BNP mRNA expression levels in the hearts of offspring male mice (a) and female mice (b). *n* = 4 mice/group; data are expressed as means ± SD and were analysed using an independent samples *t*‐test. *P < 0.05 compared with control, **P < 0.01 compared with control.

Based on the aforementioned morphological observations of hypertrophic pathological changes in mice myocardium, we further validated the myocardial hypertrophy phenotype at the molecular level by detecting the gene expression levels of atrial natriuretic peptide (ANP) and brain natriuretic peptide (BNP) in mouse cardiac tissue. Experiments revealed elevated mRNA expression levels of ANP and BNP in the hearts of male offspring following paternal sleep deprivation (Figure 6a). However, no significant changes in ANP and BNP expression were observed in female offspring (Figure 6b).

### 3.6 Effects of paternal sleep deprivation on the mechanisms of cardiac hypertrophy in offspring

**FIGURE 7:**
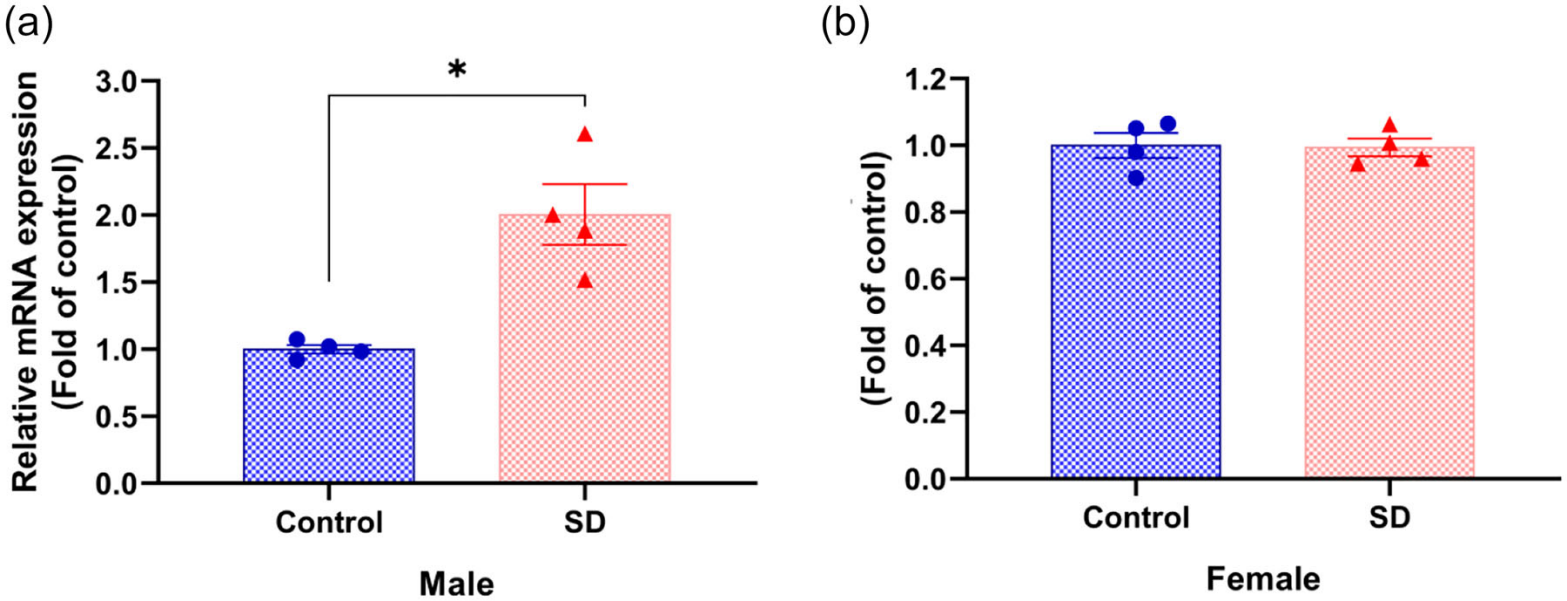
Effects of paternal sleep deprivation on mRNA expression of GATA4 expression in the heart of offspring. RT‐qPCR detection of GATA4 mRNA expression levels in the hearts of offspring male mice (a) and female mice (b). *n* = 4 mice/group; data are expressed as means ± SD and were analysed using an independent samples *t*‐test. *P < 0.05 compared with control.

To investigate the potential molecular mechanisms underlying myocardial hypertrophy in mice, we examined the expression levels of the cardiac‐specific transcription factor GATA binding protein 4 (GATA4). We found that GATA4 expression was significantly elevated in male offspring (Figure 7a), while no significant changes were observed in female offspring (Figure 7b).

## 4 DISCUSSION

A large body of evidence confirms that sleep loss is a risk factor for respiratory, cardiovascular and neurological disorders (Damigou et al., 2023; Henst et al., 2019; Salari et al., 2023) and that there is potential for transgenerational toxicity. Previously, many epidemiologists have gradually begun to focus on the transgenerational toxic effects of sleep loss. The traditional view holds that intergenerational transmission primarily involves maternal intrauterine environmental influences, such as maternal sleep deprivation during pregnancy having an impact on the offspring's cardiopulmonary system, nervous system, reproductive capacity and cognitive ability (Gerardin et al., 2005; Selle et al., 2022; Zhang et al., 2023). However, recent epigenetics research has revealed that a father's lifestyle, dietary patterns and metabolic status can also influence offspring health across generations through epigenetic mechanisms. For instance, changes in sperm small noncoding RNAs induced by a father's high‐fat diet can be transmitted to offspring via fertilization, leading to metabolic disorders in the next generation (Hernandez et al., 2024). A father's cardiovascular health score is negatively correlated with offspring cardiovascular disease through epigenetic mechanisms (Carbonneau et al., 2025). Furthermore, sleep deprivation in fathers induces metabolic disturbances in male offspring by altering DNA methylation (Zeng et al., 2023), which is closely related to this experiment. In this study, we constructed a male mouse model of acute sleep deprivation before pregnancy to investigate the changes in cardiac function in offspring mice by sleep deprivation before pregnancy at the morphological and functional levels, and to study the potential mechanism.

Body weight is one of the important indicators for measuring the overall health status. In this study, we first monitored the body weight of offspring mice after birth and found that except for offspring male mice at 6 weeks, 8 weeks and 10 weeks after birth, there was no statistically significant difference in the effect of sleep deprivation on the body weight change of offspring mice before pregnancy. Parental cadmium exposure has been found to increase the body weight of offspring mice at 4−8 weeks (Zhang et al., 2023), but the related mechanism was not clear. In addition, it has been shown that paternal bisphenol A exposure can impair cardiac development in offspring (Lombó et al., 2015), and older paternal age is also associated with an increased risk of heart failure in offspring (Ismail et al., 2022). To assess the effect of pre‐pregnancy paternal sleep deprivation on cardiac function in offspring mice as adults, we used echocardiography to examine cardiac function parameters in offspring mice. It was found that sleep deprivation of the parents before pregnancy caused increased heart rate, systolic and diastolic dysfunction in offspring male mice, accompanied by significantly increased LVAW and LVPW, suggesting myocardial hypertrophy in mice; while for females, only systolic dysfunction was shown, which was consistent with our heart tissue section observations. In general, the transgenerational toxic effects of paternal exposure on offspring showed mostly sex differences, regardless of mouse body weight or cardiac function. In addition, we observed lower sperm quality and endocrine dysfunction in a population of mice after paternal sleep deprivation, as evidenced by abnormally elevated serum testosterone levels. We hypothesize that it may act through a complex genetic and epigenetic mechanism, which together act on the developmental process of the offspring mice, ultimately leading to the development of cardiac hypertrophy.

At the molecular level, ANP and BNP are the main hormones produced by the heart (Ramos & de Bold, 2006). Not only are these hormones key players in the regulation of cardiac function and blood pressure, but they are also used in clinical practice to assess the severity of heart failure (Al‐Attas et al., 2015; Prathapan et al., 2017); they are also recognized as an important diagnostic basis for myocardial hypertrophic conditions (Dickstein et al., 1995; Jortani et al., 2004). It has been reported that phenanthrene, a polycyclic aromatic hydrocarbon, a major environmental pollutant, has been found to trigger the onset of cardiac hypertrophy, a process notably manifested by increased levels of ANP and BNP expression (Huang et al., 2016). In addition, a study on zebrafish showed that exposure to low concentrations of benzo(a)pyrene during the early stages of life led to an increase in the heart to body weight ratio, facilitated the formation of cardiac hypertrophy, which further manifested itself in adulthood as enhanced expression of ANP and BNP in the heart (Huang et al., 2014). Moreover, previous studies by our group have also shown that cardiac hypertrophy in mice is accompanied by an increase in ANP and BNP expression (Zhang et al., 2018). Accordingly, we performed an assay of ANP and BNP expression levels in mouse cardiac tissues, and the results revealed an interesting phenomenon: a state of sleep deprivation of the father prior to gestation surprisingly led to signs of cardiac hypertrophy in his offspring male mice, and this pathological change was accompanied by a significant increase in the expression of ANP and BNP in the heart.

Cardiac transcription factors play a crucial role in the development of the heart; they not only guide the normal formation of the heart, but are also deeply involved in and regulate the mechanisms that regulate the heart's response to stress (Mubeen et al., 2023). Among the many cardiac transcription factors, Csx/Nkx2.5, GATA4 and TBX5 have attracted much research attention because of their significant roles in the process of cardiac hypertrophy, and they are regarded as key regulators closely related to cardiac hypertrophy (McCulley & Black, 2012). Abnormal changes in these factors may disrupt the normal expression patterns of their downstream target genes, which in turn drive the development of cardiac hypertrophy, exacerbate the progression of heart failure, and may induce other types of heart disease (Chung & Rajakumar, 2016). A study in diabetic model mice showed that decreased expression of GATA4 in the heart altered expression of downstream genes *ANP* and *BNP* (Broderick et al., 2012), which is consistent with our findings that GATA4 expression was significantly increased in male offspring mice and modulated ANP and BNP expression in the heart.

Interestingly, in this experiment, male offspring mice subjected to paternal sleep deprivation developed cardiac hypertrophy on the surface, while female offspring mice did not develop this change, indicating that there was a sex difference in the phenomenon of cardiac hypertrophy in offspring mice induced by sleep deprivation of fathers before pregnancy. Both our team's previous findings and those of other researchers on preconception environmental exposures broadly confirm that cross‐generational toxic effects differ significantly between males and females, and that this difference is more pronounced in male offspring, showing a clear sex bias. These sex differences may be associated with sex hormone levels and sex‐specific epigenetic regulatory mechanisms (Prajapati et al., 2022). Androgens may enhance cardiac sensitivity to adverse environmental exposures in the paternal generation, promoting activation of cardiac hypertrophy‐related signalling pathways, whereas oestrogens may exert protective effects, buffering the negative consequences of paternal sleep deprivation. Furthermore, sex‐specific epigenetic modifications, such as DNA methylation and histone modifications (Bhattacharya et al., 2024), may also lead to differing gene expression patterns in offspring of opposite sexes, ultimately resulting in distinct cardiac phenotypes. The precise mechanisms underlying these effects remain to be explored. This finding not only provides new perspectives for understanding the development of cardiovascular diseases, but also provides a solid scientific basis for future prevention, early diagnosis and personalized treatment strategies for cardiovascular diseases.

Although this study has achieved certain results, it still has some limitations. The research was conducted solely in animal models, and mice differ from humans in physiological and genetic backgrounds. Further population cohort studies are needed to validate the relationship between parental sleep deprivation and offspring cardiac health, thereby providing evidence for clinical prevention and intervention. Furthermore, while this study examined changes in mRNA levels of GATA4, ANP and BNP, it did not delve into their protein expression levels, post‐translational modifications, or specific signalling pathway regulatory mechanisms. Future research is needed to comprehensively elucidate the molecular mechanisms by which acute parental sleep deprivation affects offspring cardiac function.

### 4.1 Conclusion

Overall, our results suggest that acute sleep deprivation in the father before pregnancy leads to decreased cardiac function in offspring mice, which is more significant in male mice and is accompanied by the development of cardiac hypertrophy, and the cardiac‐specific transcription factor GATA4 regulates the expression of ANP and BNP involved in the above biological processes.

## AUTHOR CONTRIBUTIONS

Writing—Original Draft, Funding acquisition: Yingying Zhang. Formal analysis: Leyao Wang. Investigation: Jie Wang. Validation: Yun Pan. Investigation: Wen Sun. Data Curation: Zhiwei Zou. Data Curation, Funding acquisition: Wenlin Bai. Resources, Funding acquisition: Ruiling Fang. Resources, Funding acquisition: Yue Zhang. Resources, Funding acquisition: Ping Zhang. Resources, Funding acquisition: Yuanbao Zhang. Supervision, Project administration, Writing—Review & Editing, Funding acquisition: Lijian Lei. Supervision, Project administration, Funding acquisition, Writing—Review & Editing: Wenping Zhang. All authors have read and approved the final version of this manuscript and agree to be accountable for all aspects of the work in ensuring that questions related to the accuracy or integrity of any part of the work are appropriately investigated and resolved. All persons designated as authors qualify for authorship, and all those who qualify for authorship are listed.

## CONFLICT OF INTEREST

The authors declare that they have no known competing financial interests or personal relationships that could have appeared to influence the work reported in this paper.

## Data Availability

The data supporting the findings of this study are available from the corresponding author upon reasonable request.
